# Targeted Forward Genetics: Population-Scale Analyses of Allele Replacements Spanning Thousands of Base Pairs in Fission Yeast

**DOI:** 10.1534/g3.119.400805

**Published:** 2019-10-09

**Authors:** Aaron J. Storey, Hsin-Ping Wang, Reine U. Protacio, Mari K. Davidson, Wayne P. Wahls

**Affiliations:** Department of Biochemistry and Molecular Biology, University of Arkansas for Medical Sciences, Little Rock, AR 72205-7199

**Keywords:** gene targeting, allele replacement, genome editing, recombination

## Abstract

Precise allele replacement (genome editing), without unwanted changes to the genome, provides a powerful tool to define the functions of DNA elements and encoded factors in their normal biological context. While CRISPR is now used extensively for gene targeting, its utility for precise allele replacement at population scale is limited because: (A) there is a strict requirement for a correctly positioned PAM motif to introduce recombinogenic dsDNA breaks (DSBs); (B) efficient replacements only occur very close to the DSBs; and (C) indels and off-target changes are frequently generated. Here we show, using a saturated mutation library with about 15,000 alleles of the *ade6* gene of *Schizosaccharomyces pombe*, that pop-in, pop-out allele replacement circumvents these problems. Two rounds of selection ensure that clones arise by homologous recombination with the target locus. Moreover, the exceptionally high efficiency allows one to carry out the process in bulk, then screen individual clones for phenotypes and genotypes. Alleles were introduced successfully throughout the region targeted, up to 1,956 base pairs from the DSB. About 11% of mutant alleles were hypomorphic, demonstrating utility for analyses of essential genes and genetic elements. This process of “targeted forward genetics” can be used to analyze comprehensively, across thousands of base pairs within a specific target region, a variety of allelic changes, such as scanning amino acid substitutions, deletions, and epitope tags. The overall approach and optimized workflow are extensible to other organisms that support gene targeting.

The components of any biological pathway, and often their order of function, can be elucidated genetically as long as mutations in that pathway produce a phenotype that can be scored. Subsequently, the biomolecules of interest (such as DNA, RNA, proteins or post-translational modifications) can be studied at high resolution using additional genetic tools, such as systematic mutagenesis, to help define their biochemical activities, functional architectures, and pathway interactions.

Extrachromosomal vectors can be manipulated easily and are therefore used frequently to study the effects of mutations in regulatory DNA sequences, RNAs and proteins [*e.g.*, (Davidson and Doranz 2014; Athanasopoulos *et al.* 2017; Mackie and Brodsky 2018)]. A process called “plasmid shuffle” provides a refinement of this approach by allowing one to replace the genomic copy with a plasmid-borne, wild-type allele; the wild-type copy is subsequently replaced with plasmid-borne mutant alleles, thus facilitating the identification and analysis of conditional mutations in essential genes [*e.g.*, (Kiely *et al.* 2000; Dacher *et al.* 2014; Sharma *et al.* 2016)]. Unfortunately, in both cases the experimental approach can introduce artifacts which confound the results.

First, episomal vectors lack the surrounding chromosomal context that facilitates, and can even be essential for, the function of some genomic elements. Examples of such chromosomal context-dependent regulation include those for epigenetic inheritance (Wang and Moazed 2017), replication origins (Pratihar *et al.* 2015), DNA damage repair (Wyrick and Roberts 2015), long-range transcription regulators (Catarino *et al.* 2017), centromeres (Romeo *et al.* 2016), and meiotic recombination hotspots (Wahls and Davidson 2012). Second, the increased copy number of genes in extrachromosomal vectors, or the use of heterologous promoters in single-copy plasmids, typically alters protein expression levels (Chino *et al.* 2013). As exemplified by a study in which 99 wild-type transcription factors were overexpressed, the majority of such non-physiological expression levels produce deleterious phenotypes (Vachon *et al.* 2013). Reciprocally, even modest reductions in the expression of wild-type proteins can also produce “mutant” phenotypes, as exemplified by haploinsufficiency (Moris *et al.* 2016; Bae *et al.* 2017). Phenotypes caused by non-physiological context, dosage or expression levels of extrachromosomal vectors complicate interpretation of results and can even yield false-positive or false-negative results.

An alternative approach for sequence-*vs.*-function studies involves using precise allele replacement (via homologous recombination) to introduce mutations into the chromosome at the endogenous locus, where the mutant alleles are propagated stably at the correct dosage and are expressed in native context from their normal regulatory elements (Grimm *et al.* 1988; Mudge *et al.* 2012; Gao *et al.* 2014). This eliminates confounding effects of incorrect dosage or aberrant expression and, moreover, supports the mutational dissection of elements that only function properly in their native chromosomal context. Unfortunately, precise allele replacement—in which there are no additional chromosomal changes—is labor intensive and, in most organisms, relatively inefficient. While CRISPR is now used extensively to target genes, its utility for allele replacement through homologous recombination is limited to short stretches of homology around CRISPR-catalyzed DNA breaks and by additional factors, such as low efficiency of homologous replacement *vs.* non-homologous end joining and the generation of off-target scars (Ford *et al.* 2019; Findlay *et al.* 2014; Lamb *et al.* 2017). Alternative approaches that were developed to make allele replacement more facile, such as PCR-based gene targeting (Bähler *et al.* 1998; Krawchuk and Wahls 1999) and recombinase-mediated cassette exchange (Thomson and Ow 2006; Watson *et al.* 2008; Turan *et al.* 2011), can be efficient and are thus extensible to high throughput screening. However, these approaches are imprecise in that they place additional changes into the chromosome (such as heterologous promoters or terminators, selectable marker cassettes, or recombination signal sequences), each of which can affect the function of the targeted locus or expression levels of the factors that it encodes.

Here we describe a powerful new methodology for precisely targeted, saturating mutational analyses of discrete chromosomal elements *in situ*. More than 100,000 independent allele replacements, distributed over long regions of the target locus (thousands of base pairs), can be generated simultaneously in each experiment. The approach efficiently generates and identifies functionally null and hypomorphic mutations throughout the region targeted. This process of population-scale, “targeted forward genetics” over large distances allows scientists to rapidly dissect the structure and function of specific chromosomal elements and their encoded factors under native, biologically relevant conditions.

## Materials and Methods

### Culture methods

Fission yeast strain WSP588 (*h+ ura4-D18*) was grown in rich YEL media containing 5 g/L yeast extract (Sunrise Scientific) and 3% (w/v) glucose, or minimal NBL media containing 1.7 g/L yeast nitrogen base (Difco), 5 g/L ammonium sulfate, and 1% (w/v) glucose. The media were supplemented if required with adenine, uracil (each 100 µg/ml) or 5-fluoroorotic acid (FOA, 2 mg/ml). Solid media (YEA and NBA) included 2% (w/v) Difco agar.

### Construction of targeting vector and PCR-based mutagenesis

The gene targeting vector was constructed by first sub-cloning the *ura4^+^* gene, on a 1.8 kbp *Hin*dIII fragment, into pBluescript II KS (-). The *ade6^+^* locus (consisting of 2500 bp 5′ homology, the *ade6^+^* ORF, and 800 bp 3′ homology) was then inserted by sub-cloning into the plasmid’s multiple cloning site. The restriction sites *NotI* and *PstI* were added to the 5′ and 3′ ends of the insert by PCR to facilitate directional cloning. The resulting plasmid contains a unique *SpeI* restriction site near the center of the 800 bp 3′ homology region. The plasmid DNA was sequenced to confirm its integrity and eliminate any clones with spurious mutations.

Mutagenic PCR (Dymond 2013) was performed using Taq polymerase (NEB) and the following PCR conditions. Each 50 µL reaction contained 0.2 mM dNTPs, 10 mM Tris-HCl pH 8.8, 50 mM KCl, 0.5 µM of each primer (forward primer ade6-pr7, 5′-TTTTTCAACATTTACCATCTCA-3′; reverse primer ade6-pr20, 5′-TCCTACAGCTATATGCGTGATTAC-3′), 1 ng template plasmid DNA, 4 U polymerase, 250 µM MgCl_2_ and 240 µM MnCl_2_. The thermal cycle steps consisted of one round of denaturation at 95° for 2 min; 40 cycles of 95° for 30 sec, 65° for 1 min, and 72° for 4 min; and a final extension step of 72° for 10 min. Following mutagenesis, the amplified *ade6* DNA was digested with restriction endonucleases *Bam*HI and *Spe*I, was gel purified, and was subcloned into the corresponding position of the gene targeting vector.

### Transformation and selection of allele replacements

To generate a high-titer gene targeting library, 50 small scale transformations of *E. coli* were performed in parallel using the OpenWetWare TOP10 protocol, 2 µl of DNA, and 50 µl of TOP10 competent cells. Following recovery in SOC media, the transformation mixtures were combined and an aliquot was plated in serial dilutions on LB-ampicillin to determine the library titer. The rest of the transformation mixture was grown in LB-ampicillin liquid and was used to prepare library DNA. Additional aliquots of transformed cells were frozen in glycerol stocks (25% final concentration) and stored at -80° to enable regrowth of the library.

Yeast transformations were performed using the lithium acetate procedure. Cells were grown to a density of 0.5 × 10^7^ cells/ml in NBL plus uracil and harvested by centrifugation. Pellets were washed three times with sterile water and resuspended in 100 mM lithium acetate (pH 4.9) at a cell density of 1 × 10^9^ cells/ml. Aliquots of cells (100 µL) were mixed with 1 µg of linearized plasmid, 290 µL of 50% PEG 3350 in 100 mM lithium acetate (pH 4.9), and incubated for 1 hr at 25°. Cells were heat-shocked for 30 min at 42°, cooled to room temperature, then inoculated into 10 ml of YEL media with 100 µg/ml uracil and incubated at 32° for 1 hr for recovery. To determine the transformation efficiency and the titer of transformants, aliquots of transformed cells were plated in serial dilutions on selective agar plates (NBA without uracil). For large scale screening, transformations were carried out in parallel, pooled, and subjected to additional steps as described in the Results section and figures. In brief, the transformed cells were cultured sequentially three times in 1,000 ml of NBL without uracil and once in 100 ml of YEL supplemented with uracil and adenine. Then, serial dilutions were plated on YEA with FOA. Candidate *ade6* mutants were identified by their characteristic pink or red colony color on YEA; mutant phenotypes were confirmed by serial dilution plating assays using minimal NBA with uracil and that contained or lacked adenine.

### Sequencing

Mutations within the gene targeting vector library and in the *ade6* locus of fission yeast cells with an Ade^-^ phenotype following allele replacement were identified by the UAMS DNA Sequencing Core Facility. Template DNAs were from individual plasmid clones of the library and from PCR amplifications of genomic DNA from individual yeast colonies, respectively; primers were ade6-pr7 and ade6-pr20 (described above) and ade6-pr4 (5′-GCAATAATCACACGCAACCCTCTT-3′). Sequencing reactions were carried out with a 3500 Genetic Analyzer according to the instructions of the manufacturer (Applied Biosystems).

### Data availability

Yeast strains and other material generated by this study are available upon request. All data supporting the conclusions of this study are available within the paper and its supplemental material file. Supplemental material available at figshare: https://doi.org/10.25387/g3.9936893.

## Results

### Approach for precise and efficient allele replacements

**Figure 1:**
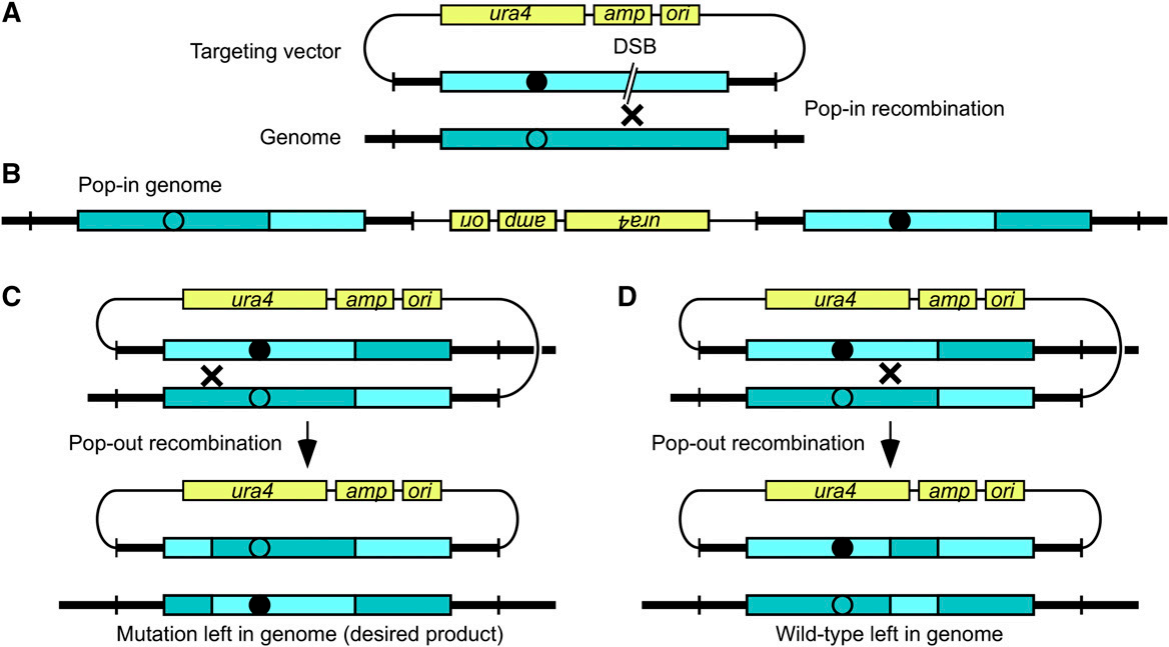
Precise allele replacement by pop-in, pop-out homologous recombination. Diagram shows how a mutation or other modification (*closed circle*) in the gene-targeting vector replaces wild-type information (*open circle*) at the target locus, such as an ORF (*blue boxes*). (A) The portion of the vector without homology to the genome (*thin line*) includes the *amp* and *ori* elements to support propagation in *E. coli* and the *ura4^+^* gene to support positive and negative selection in *S. pombe* (*yellow boxes*). The region of the vector with homology to the genomic target (*thick line*) includes the element being targeted. Cells lacking the *ura4^+^* gene (*ura4-D18*) are transformed with vector that has a recombinogenic dsDNA break (*DSB*) in the region of homology and selection is applied for uracil prototrophy. Since the vector does not have a fission yeast origin of replication (*ARS*), Ura^+^ transformants arise from recombination (×) with the genome to produce a tandem copy (B) with one wild-type and one mutant allele. (C, D) Homologous recombination (×) between tandem repeats excises the vector from the genome. Since the excised vector lacks an *ARS*, it cannot replicate and is lost during cell divisions. Removal of selection for uracil prototrophy allows the pop-out cells to survive, and subsequent plating on media with FOA selects for cells that have lost the vector. Recombination (excision) events to one side of the DSB leave the mutation in the genome (C), whereas recombination (excision) events to the other side leave the wild-type allele in the genome (D).

The mechanisms and utility of pop-in, pop-out allele replacement via homologous recombination (Gao *et al.* 2014; Køhler *et al.* 2015; Uchiyama *et al.* 2015; Folco *et al.* 2017; Laboucarié *et al.* 2017; Jørgensen *et al.* 2018; Watts *et al.* 2018) form the basis for the process of population-scale, targeted forward genetics described here. There is considerable latitude in the structure and construction of the circular gene targeting vector that is used for precise allele replacement and the only requirement is that it contains the following three components (Figure 1A).

First, the targeting vector has an *E. coli* origin of replication and selectable marker to support its propagation in bacteria. Second, it contains one or more genes to support positive and negative selection in the organism being modified. For fission yeast we use the *ura4^+^* gene, which encodes orotidine-5′-phosphate decarboxylase (Bach 1987), for both positive and negative selection (Grimm and Kohli 1988). Cells expressing *ura4^+^* can be selected for on media lacking uracil and, because 5-fluoroorotic acid (FOA) kills cells that are *ura4^+^*, FOA selects for cells that lack a functional *ura4^+^*. The third part of the vector contains a region of homology to the target locus in the genome. The homologous DNA in the targeting vector can be modified in a variety of ways, such as to contain a mutation, insertion, deletion, or addition of an in-frame epitope tag (Gao *et al.* 2014; Køhler *et al.* 2015; Uchiyama *et al.* 2015; Folco *et al.* 2017; Laboucarié *et al.* 2017; Jørgensen *et al.* 2018; Watts *et al.* 2018). The various types of potential changes (alleles) are all functionally equivalent with regard to mechanisms and efficiencies of pop-in, pop-out allele replacement (Gao *et al.* 2014).

The steps of precise allele replacement are depicted in Figure 1. The gene targeting vector is digested with a restriction endonuclease to introduce a recombination-initiating dsDNA break (DSB) within the region of homology. The linearized vector is transformed into cells of genotype *ura4-D18* (which has a 1.8 kbp deletion of the endogenous *ura4* locus) and cells are plated on media lacking uracil. Since the targeting vector lacks a fission yeast origin of replication, uracil prototrophic (Ura^+^) colonies arise from pop-in recombination events that generate a tandem duplication of the target locus, one wild-type and one mutant, flanking the *ura4^+^* cassette and other portions of the plasmid backbone (Figure 1B). Prototrophic colonies can also arise by non-homologous integration elsewhere (Davidson *et al.* 2004b), but because these lack the tandem copies of the target locus required for pop-out recombination, they will be selected against by subsequent steps of the process (Gao *et al.* 2014).

Pop-in recombinants harbor two copies of the target locus, one wild-type and one mutant, flanking the *ura4^+^* cassette (Figure 1B). These are inherently unstable because recombination between the two direct repeats causes the targeting vector to pop-out of the genome (Figure 1C-1D). Since the vector lacks a fission yeast origin of replication, it cannot be maintained as an episome and is lost in subsequent cell divisions. Thus, if one takes a Ura^+^ colony from transformation and passages it under non-selective conditions (typically one passage through liquid culture media that contains uracil), cells with pop-out recombination events can accumulate. Subsequent plating on solid media that contains FOA kills cells that remain *ura4^+^*, thus selecting for pop-out recombinants.

While the position of pop-in recombination is dictated by the position of the DSB in the gene targeting vector, pop-out recombination events can occur anywhere within the region of homology between the duplicated (direct repeat) target segments. Pop-out recombination events that occur on one side of the allelic modification will release the modified allele in the targeting vector (which is subsequently lost), and these “non-productive” events will leave the wild-type allele in the chromosome (Figure 1D). Pop-out recombination events on the opposite, “productive” side of homology will leave the modified allele in the chromosome (Figure 1C).

With regard to these flanking homologies, two key factors are germane to rational, evidence-based design of the gene targeting vector. First, the location of the DSB that is used for the pop-in step dictates whether the homology flanking the allele at the pop-out step is on the productive or non-productive side of that allele. Pop-out recombination events that occur on the DSB side of flanking homology are invariably non-productive (*e.g.*, Figure 1D). Pop-out recombination events that occur on the non-DSB side of homology are invariably productive (*e.g.*, Figure 1C). Second, the frequency with which the modified allele is left in the genome is directly proportional to the homology length ratios (Gao *et al.* 2014). For example, if 75% of the homology is on the productive (*i.e.*, non-DSB) side of the modification, then about 75% of pop-out recombination events will leave the modified allele in the chromosome. We took advantage of this property when designing our gene targeting vector (described below).

### Rationale for population-scale, targeted forward genetics

**Figure 2:**
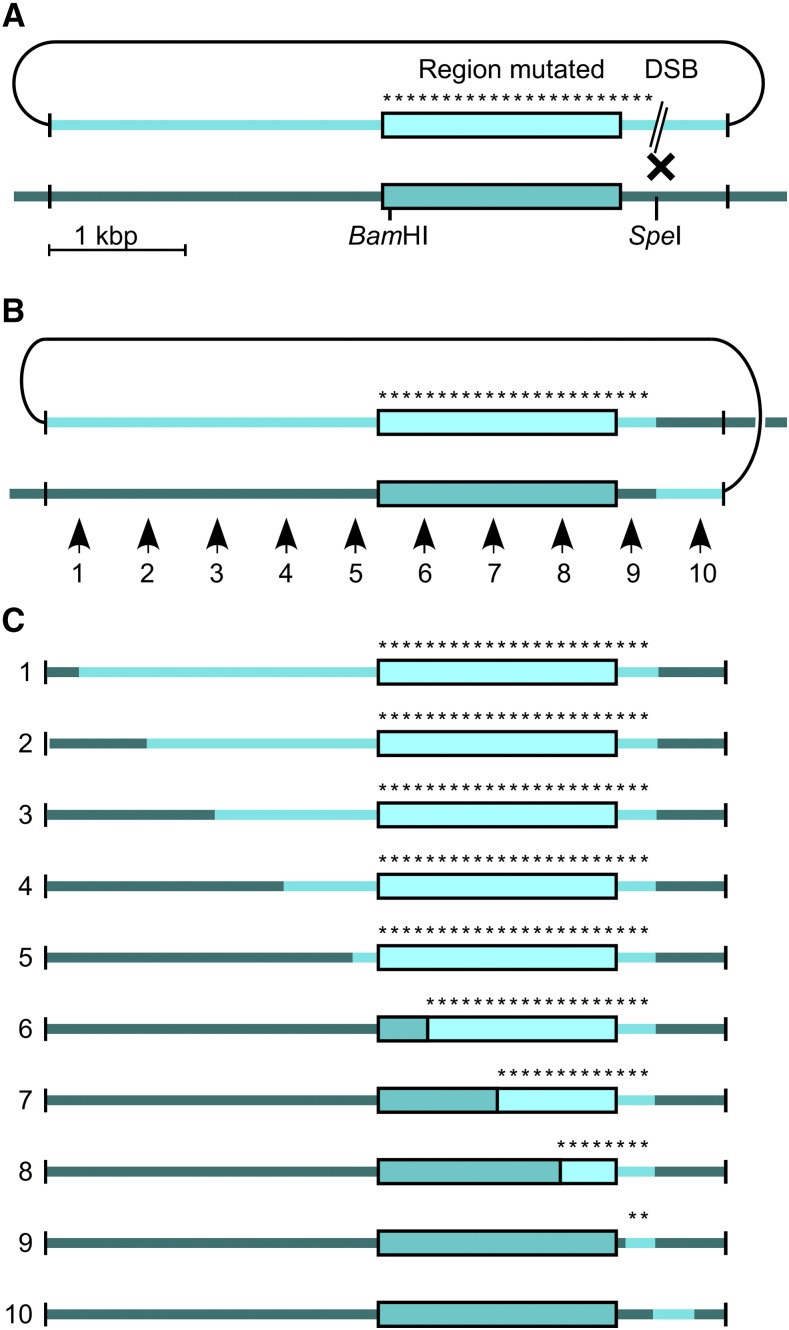
Approach for population-scale, precise allele replacements spanning thousands of base pairs of the target locus. The extent of homology (*thick lines*), *ade6* ORF (*boxes*), and position of the DSB are drawn to scale; elements are color coded for visual reference. Ideally, each DNA molecule in the targeting vector library will have a single mutation (*asterisk*) within the region of interest; the diagram illustrates their population-average distribution in the library. (A) A DSB promotes homologous recombination between the vector and genome. (B) Each pop-in recombination event yields a tandem duplication of the target locus. Subsequent pop-out recombination events can occur anywhere within the region of homology (*e.g.*, *numbered arrows*). (C) Population-average distribution of each mutation in the genome after pop-out recombination (numbers correspond to pop-out events in panel “B”). Individual clones will have only one or a few of the mutations depicted in each row, depending on the density of mutations in the gene targeting vector library (see Figure S1 for discrete examples). Note that the likelihood of leaving a given mutation in the genome is proportional to the fraction of homology on the productive (*i.e.*, non-DSB) side of that mutation. This property can be used to optimize the design of targeting vectors, as we did in this case.

One can carry out both steps of pop-in, pop-out allele replacement without having to genotype cells at any stage of the process, then screen the resulting colonies for mutant phenotypes (Gao *et al.* 2014). The majority of Ura^+^ colonies are generated by homologous recombination with the desired target locus. Because off-target, non-homologous integration events (also Ura^+^) lack the tandem copies of the target locus required for pop-out recombination, they yield Ura^-^ (FOA^r^) segregants at very low rates. Only the correctly targeted integrations (*i.e.*, those that contain tandem repeats) can undergo pop-out recombination to produce at high frequency FOA^r^ colonies (≥ 50-fold observed difference, relative to spontaneous mutations in the *ura4^+^* cassette or elsewhere) (Gao *et al.* 2014). Thus, even if the frequency of non-homologous integration is relatively high, the powerful selective advantage for FOA-resistant clones stemming from the correct integration structures (*i.e.*, targeted tandem duplications) ensures that the vast majority of FOA^r^ colonies arise via pop-in, pop-out gene targeting at the locus of interest. These factors suggested that the process could be carried out at population scale, in bulk, using a library of gene targeting vectors (Figure 2).

### Choice of target locus and design of targeting vector

We chose the *ade6^+^* locus as a model with which to develop the process of population-scale, targeted forward genetics for two reasons. First, the efficiency of pop-in, pop-out allele replacement at *ade6^+^* is similar to that of all other loci tested (Gao *et al.* 2014), so *ade6^+^* provides a representative model locus. Second, *ade6* mutants have easily scored phenotypes (Gutz *et al.* 1974). On media with limiting amounts of adenine, wild-type cells form white colonies, whereas *ade6* mutants form pink or red colonies. (The amount of pigment varies by allele, by the type of growth media used, and by the amount of adenine in that media.) Thus, one can score visually the phenotypes of hundreds of colonies on each plate. Wild-type cells also plate efficiently on media lacking adenine, whereas *ade6* mutants are auxotrophic, which provides a second, independent way to score mutant phenotypes.

Our goal was to test whether a population of gene targeting vectors, each containing one or a few mutations within the *ade6* ORF (1,659 base pairs), could be used for pop-in, pop-out allele replacement and high throughput (population-scale) phenotyping without prior knowledge of genotype. We therefore designed the *ade6* gene targeting vector in a way to optimize the recovery of productive pop-out recombination events. This was achieved by making the length of homology on the productive side of the *ade6* ORF longer than the length of homology on the DSB side of the ORF (Figure 2). Based on the known effects of homology length ratios (Gao *et al.* 2014), between 50% and 83% of the pop-out events should leave a mutation in the genome, depending on where the given mutation resides in the *ade6* ORF (depicted schematically in Figure 2C; see also Supplemental Material, Figure S1).

### Construction of gene targeting vector library

**Figure 3:**
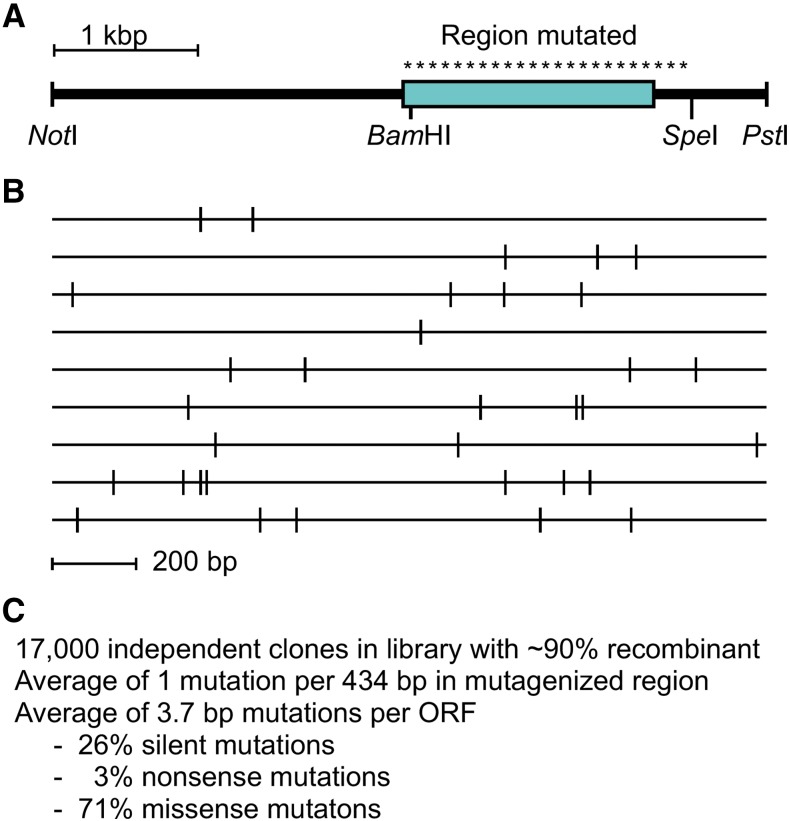
Characteristics of mutated gene targeting vector library. (A) Diagram shows portion of the *ade6* gene targeting vector with homology to the fission yeast genome, position of *ade6* ORF (*box*), and engineered restriction sites (*Not*I and *Pst*I) used for cloning into the vector. Mutagenic PCR and sub-cloning were used introduce mutations (*asterisks*) between the *Bam*HI and *Spe*I restriction sites, encompassing essentially all of the *ade6* ORF and part of its 3′ region. (B) Distribution of mutations within the *ade6* ORF (1,659 base pairs) of individual clones from the library for which full-length DNA sequence information was obtained. (C) Characteristics of gene targeting vector library and mutations therein. For DNA sequences of individual clones see Figure S2 and for their encoded protein sequences see Figure S3.

We first constructed a gene-targeting vector that harbors a wild-type *ade6^+^* locus. A portion of the vector encompassing the *ade6* ORF was then amplified by mutagenic PCR (Dymond 2013), was subcloned directionally back into the vector (Figure 3), and the resulting library was transformed into *E. coli*. A small aliquot of the transformation mixture was diluted serially and plated on LB-amp plates to determine the library titer; the majority of the transformation mixture was expanded in liquid culture and was used to produce the gene-targeting library DNA. The library contained approximately 17,000 independent clones and, based on restriction mapping of individual clones, about 90% of these were recombinant.

The *ade6* inserts within individual recombinant clones of the library were sequenced to determine the efficiency of mutagenesis and the distribution of mutations. Each of the isolates analyzed contained a different pattern of mutations (Figure 3 and Figure S2), demonstrating that they were independent clones. On average, there was one mutation per 434 base pairs within the mutagenized region and these were apparently distributed stochastically. All of the identified mutations were single base pair substitutions which, upon translation, would either be silent (26% of mutations), encode a stop codon (3%), or lead to an amino acid substitution (71%) (Figure 3 and Figure S3). Each clone contained on average 3.7 base pair mutations (encoding 2.7 protein mutations) within the *ade6* ORF and, statistically, there was a greater than 97% probability that each recombinant clone contained at least one mutation in the ORF. Based on the library titer, recombinant fraction, mutation density and length of the *ade6* ORF, each base pair in the ORF was mutated on average about 10 times in the library. We conclude that the library was saturated for mutations and suitable for targeted allele replacement.

### Population-scale, targeted forward genetics spanning thousands of base pairs of the target locus

The process of allele replacements in bulk using tens of thousands of DNA clones (Figure 2) is similar to the process of allele replacement using individual DNA clones (Figure 1). There is, however, one important difference. For pop-in, pop-out allele replacement using individual DNA clones, Ura^+^ transformants are selected for by colony growth on solid media lacking uracil, which effectively eliminates any non-transformed (Ura^-^) cells. However, to efficiently carry out such screens for tens of thousands of DNA clones simultaneously, intermediate steps of the process must be carried out in bulk using liquid cultures. This is a potentially confounding factor because during transformation only a small fraction of the cells become transformed successfully to Ura^+^. The vast majority of cells remain Ura^-^ and, while they do not divide in liquid culture under selection for uracil prototrophy, they might remain viable and emerge from quiescence when that selection is removed to permit pop-out recombination. Upon subsequent selection for resistance to FOA those cells, which had not experienced allele replacement, might resume growth, effectively reducing the frequency with which the desired phenotypes (*i.e.*, from successful allele replacements) are observed at the end of the screen.

**Figure 4:**
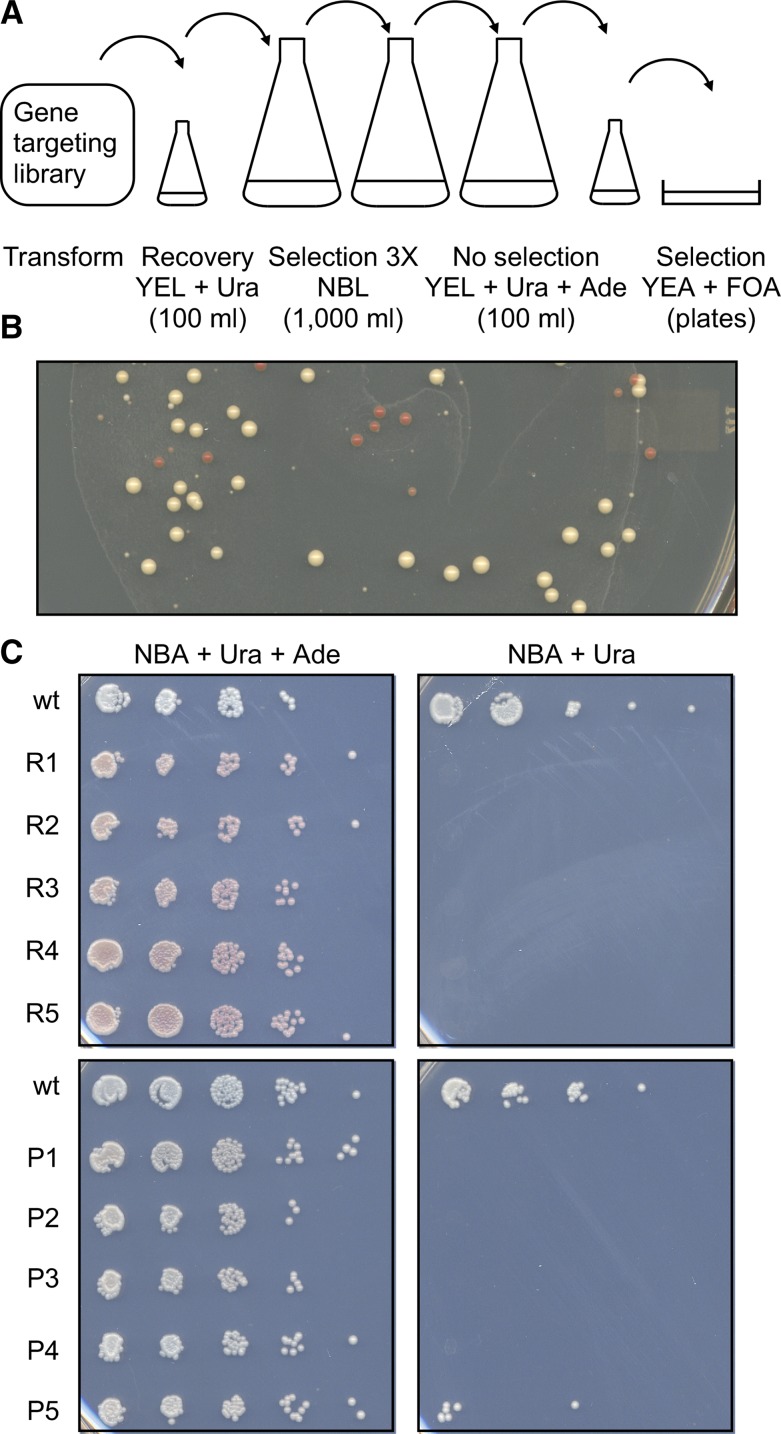
Optimized workflow for targeted forward genetics. (A) The gene targeting vector library is transformed into cells, which are then cultured briefly in liquid media that contains uracil to permit expression of Ura4^+^ protein. A small aliquot is then plated in serial dilutions on solid media lacking uracil to determine the titer of Ura^+^ transformants (not shown). Three sequential passages in liquid media lacking uracil selects for Ura^+^ transformants and against non-transformed Ura^-^ cells. One passage in media that contains uracil (and, for our screen, adenine) allows pop-out recombinants (Ura^-^ and potentially Ade^-^) to accumulate. Subsequent plating of serial dilutions on solid media that contains FOA selects for the pop-out recombinants (Ura^-^ FOA^r^). These are then screened for their phenotype(s) of interest. (B) Example of direct screen for *ade6* mutant phenotype. The FOA-containing media (YEA) has limiting amounts of adenine. On such media, *ade6^+^* cells form white colonies and *ade6* mutants form pink or red colonies. (C) Confirmation of mutant phenotypes. Serial dilutions of wild-type and presumptive *ade6* mutants from the colorimetric screen (R, red; P, pink) were plated on minimal media that contains or lacks adenine. Each candidate that was analyzed passed this secondary test, although some mutants were hypomorphic (*e.g.*, P5, see also Figure 5). In every case tested, subsequent DNA sequencing of the *ade6* target locus confirmed that each mutant clone harbored one or more coding mutations in the *ade6* ORF (Figure S4).

To address this issue, we determined empirically how many passages, under selection for uracil prototrophy, were required to essentially eliminate any viable, non-transformed (Ura^-^) cells from liquid cultures that contained a mixture of Ura^-^ and Ura^+^ cells. This step was incorporated into the overall protocol and workflow (Figure 4), as follows.

#### Population-scale transformations (pop-in step):

DNA from the gene targeting vector library, which contained about 17,000 independent clones, was digested with restriction endonuclease *Spe*I to introduce a recombination-promoting DSB within the region of homology (Figure 2A). All subsequent steps of the optimized process are depicted in Figure 4. The library was transformed into fission yeast and the cells were incubated for 1 hr in 100 ml of YEL (rich) media supplemented with 100 µg/mL of uracil to permit the cells to recover from the shock of transformation and begin expressing Ura4 protein. A small aliquot of the transformation mixture was diluted serially and plated on NBA (minimal) media lacking uracil to determine the number of Ura^+^ colonies. This revealed, after adjustment for dilution factors, that there were about 164,000 total transformants. This number of transformants was nearly ten-times the number of individual DNA clones in the library (∼17,000), ensuring with high probability that each DNA clone from the library was represented in the population of transformants.

#### Selection for Ura^+^ transformants:

Cells from the transformation mixture were inoculated into 1,000 ml of NBL media lacking uracil. The culture was incubated until it reached a density of about 3 × 10^7^ cells per ml. Approximately 1 × 10^9^ cells were transferred into 1,000 ml of fresh media and were cultured to a final density of about 3 × 10^7^ cells per ml. This process was repeated another time (for a total of three passages), allowing the population of transformed (Ura^+^) cells to expand exponentially, while simultaneously reducing the titer of (*i.e.*, number of viable) non-transformed (Ura^-^) cells. Because the number of cells inoculated into each sequential culture (about 1 × 10^9^) vastly exceeded the number of original transformants (∼164,000), this process ensured that there were no bottlenecks and that there was a high probability that each original Ura^+^ clone was represented in the final population.

#### Removal of selection (pop-out step):

Approximately 1 × 10^8^ cells were transferred into 100 ml of rich YEL media supplemented with uracil and adenine and were grown to a final density of about 3 × 10^7^ cells per ml. Under these non-selective conditions, cells that are rendered Ura^-^ by pop-out recombination events accumulate in the culture; the additional adenine ensured that any *ade6* mutants that arise in the population can grow efficiently.

#### Selection for pop-out recombinants:

Serial dilutions of cells were plated on solid YEA media that contained FOA. The presence of FOA selected for Ura^-^ cells that had successfully undergone pop-in, pop-out recombination. Such FOA^r^ colonies can be scored for any phenotype of interest, which in our case included phenotypes associated with mutations in the target locus *ade6*.

#### Scoring of mutant phenotypes:

Because YEA plates contain limiting amounts of adenine, we could directly identify—on the same plates that were used to select for pop-out recombinants—colorimetric phenotypes that are characteristic of *ade6^+^* (white) and *ade6* mutant (pink or red) colonies (Figure 4B). About 15% of the FOA^r^ colonies exhibited an *ade6* mutant phenotype based on colony color. Candidate mutants were retested by plating serial dilutions of cells on minimal media. In each case, the presumptive *ade6* mutants grew as well as wild-type cells on media that contained adenine, but either failed to plate or plated inefficiently on media lacking adenine (Figure 4C), indicating that the individual isolates have no obvious growth defects other than having become auxotrophic or hypomorphic for adenine.

#### Confirmation of mutations in the target locus:

We sequenced the *ade6* locus from a collection of clones that had an *ade6* mutant phenotype. In every case, there were mutations at the chromosomal *ade6* target locus and these occurred at various positions along the region of the gene that was originally mutated in the gene targeting vector library (Figure S4). We conclude that the population-scale allele replacement process is precise and systematic, as well as efficient (above), allowing one to carry such screens to saturation.

### High frequency of hypomorphic mutations

**Figure 5:**
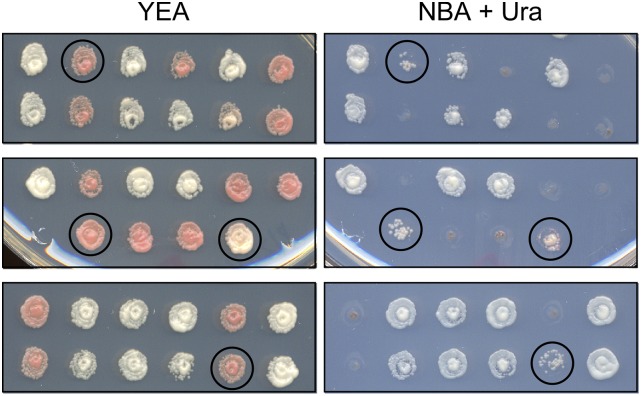
Targeted forward genetics efficiently generates and identifies hypomorphic mutations. Suspensions of cells from randomly selected white, red and pink colonies from the initial screen (n = 500) were spotted onto YEA (which contains limiting amounts of adenine) and NBA without adenine. Regions of interest from three different pairs of plates are shown. The red or pink color on YEA is diagnostic for an *ade6* mutation and failure to grow on NBA is diagnostic for a complete loss-of-function mutation. A subset of the mutants (11%) exhibited a hypomorphic phenotype (*circles*) of intermediate growth in the absence of adenine.

While the majority of presumptive *ade6* mutants from the colorimetric screen were auxotrophic for adenine (*i.e.*, functionally null) when retested by serial dilution plating assays, a subset of candidates were hypomorphic in that they exhibited some growth in the absence of adenine (*e.g.*, Figure 4C). To explore this further, we selected at random white (presumptive wild-type), red and pink (presumptive mutant) colonies from the primary screen. Aliquots of cells from those clones were spotted onto YEA media with limiting adenine and NBA minimal media without adenine (Figure 5). All clones plated efficiently on YEA and their colony colors from the initial screen were recapitulated. The presumptive wild-type cells grew efficiently in the absence of adenine, confirming that they were either wild-type for or harbored silent mutations within *ade6*. Most of the presumptive mutants failed to plate in the absence of adenine, demonstrating that they were fully auxotrophic. However, about 11% of the mutants exhibited an intermediate level of growth in the absence of adenine (Figure 5), demonstrating that they were hypomorphic. In each case sequenced, the auxotrophic and hypomorphic mutants harbored mutations in the *ade6* ORF (Figure S4). We conclude that targeted forward genetics can generate and reveal hypomorphic mutations within the target element of interest, which is of particular utility for analyses of essential genes and genetic elements (see Discussion).

### Population-scale, targeted forward genetics is robust and reproducible

We repeated the entire process, from construction of the gene targeting vector library through to the analyses of fission yeast clones following pop-in, pop-out homologous recombination. The frequency with which mutant phenotypes were revealed in the second screen was similar to that from the first screen and to frequencies obtained in pilot experiments conducted during optimization of the process. In each case, about 10–15% of FOA^r^ colonies had a mutant phenotype. We conclude that the overall approach is reproducible, as well as robust (*i.e.*, yields mutants at high frequency).

## Discussion

### A process for saturating mutational analyses of discrete genomic elements in situ

The targeted forward genetics approach developed and validated in this study combines targeted allele replacement with forward genetics. The targeting stage uses homologous recombination to sprinkle mutations (or any other specific changes desired), in high throughput fashion, into the discrete chromosomal target element of interest. Importantly, the pop-in, pop-out allele replacement introduces only the desired changes into the genome, without any additional changes at the target locus or elsewhere (Gao *et al.* 2014). Moreover, as shown in this study, the allele replacements can be introduced efficiently at population scale over long regions of the target locus (thousands of base pairs) that are remote from the recombination-initiating DSB. The subsequent, forward genetics step screens rapidly, at the scale of large populations, for mutant phenotypes without prior knowledge of mutation type.

The process of targeted forward genetics is straightforward, relatively inexpensive and very efficient. Starting with a gene targeting library in which each clone contained, on average, 3.7 base pair-substitution mutations in the *ade6* ORF (an average of 2.7 nonsynonymous mutations per ORF) (Figure 3 and Figures S2-S3), about 15% of the resulting fission yeast colonies exhibited a mutant phenotype (Figure 4). The process is also precise because in every case tested the mutant phenotypes arose from mutations introduced into the locus that was targeted (Figure S4). The exceptionally high mutant discovery rate (15% of colonies) is even more impressive when one considers that pop-in, pop-out allele replacement can leave either a wild-type or mutated allele in the genome (Figure 1), that a subset of the mutations left in the genome are silent translationally (Figure 3), and that not all missense mutations will compromise a given protein’s biochemical activities. Together, these findings demonstrate that targeted forward genetics can be used for saturating mutational analyses of specific genomic elements *in situ*.

### A caveat about origins of replication

Our population-scale approach employs an allele replacement method (Gao *et al.* 2014) that has been used successfully in fission yeast for scores of individual gene targeting vector constructs [for examples, see (Davidson *et al.* 2004a; Gao *et al.* 2008; Kan *et al.* 2011; Gao *et al.* 2013; Gao *et al.* 2014; Uchiyama *et al.* 2015; Køhler *et al.* 2015; Folco *et al.* 2017; Laboucarié *et al.* 2017; Jørgensen *et al.* 2018; Bronstein *et al.* 2018; Watts *et al.* 2018; Okita *et al.* 2019)]. This method involves gene targeting vectors that lack an origin of replication (Figure 1). We note, for the following reasons, that placing an origin of replication within the region of homology might reduce the overall efficiency of targeted forward genetics.

Linear, origin-containing DNA molecules can circularize, and, consequently, can replicate as extrachromosomal plasmids (Grimm and Kohli 1988; Szankasi *et al.* 1988). The linear, origin-containing vectors can also integrate into the genome at the desired target locus by pop-in recombination events. Each type of transformation event would yield Ura^+^ colonies upon positive selection for the presence of *ura4*. Moreover, in each case, subsequent negative selection (for resistance to FOA) selects for Ura^-^ cells, regardless of whether the transformed, linear DNA had undergone pop-in, pop-out recombination or arose via circularization without gene targeting. The relevant difference is that the former would leave the desired allele replacements in the genome, whereas the latter would not. Hence, at population scale, the efficiency of allele replacements in the genome for origin-containing gene targeting vectors would be influenced by the relative rates with which the linear DNA fragment integrates in the genome or, alternatively, circularizes to form an extrachromosomal, replicating element (plasmid).

The relative rates at which these events occur have not yet been reported, so it is not known whether including an origin in the vector would have a minor or major impact on the efficiency of targeted forward genetics. Nevertheless, investigators who are interested in using the approach should be aware of the implications. First, to the extent possible, we recommend that the region of homology in the gene targeting vector should not contain an origin of replication. Second, for cases in which the region of homology must contain an origin, it might be prudent to mutate that origin to render it non-functional.

### Targeted forward genetics of essential genes

While we used a non-essential gene model to optimize the workflow and validate the process, it also provides a powerful tool to analyze essential genes and genetic elements. For modifying essential genes, the gene targeting vector can be constructed such that, after pop-in recombination, the tandem copies of the target locus will contain one full-length, wild-type copy (as depicted in Figure 1A-1B) and thus support biological activity (Gao *et al.* 2014). Pop-out recombination leaves either a wild-type or a mutant copy in the genome (Figure 1C-1D). Thus, if all pop-out isolates (from a given tandem integrant) have a wild-type copy in the genome and none have the mutated copy, this is diagnostic for lethality of that mutation [for examples see (Jørgensen *et al.* 2018; Watts *et al.* 2018)]. This straightforward diagnostic test obviates the need for more labor-intensive tests, such as constructing heterozygous mutations in diploids and following the segregation patterns of alleles through meiosis. Such diagnostics can be used for a variety of mutations, such as those that ablate an ORF, substitute amino acids within proteins, or truncate lncRNAs.

We discovered that targeted forward genetics provides a remarkably efficient way to generate and identify hypomorphic mutations (Figure 5), which is particularly useful for studying essential genes and genetic elements. Among mutant candidates from the primary screen, 11% contained hypomorphic mutations. Such mutants can be identified directly by morphological differences in colony formation (exemplified in Figure 4), by changes in cellular growth characteristics (like those shown in Figure 5), or by screening for conditional phenotypes such as temperature sensitivity. Investigators who work with essential genes or genetic elements can thus use population-scale, targeted forward genetics in saturating screens to discover numerous hypomorphic or conditional alleles with which to define biological functions. This approach could be used productively, for example, to help define the functions of “high priority” genes, which are essential genes of undefined function that are broadly conserved (often in single copy) across diverse taxa (Wood *et al.* 2019).

### Diverse libraries for diverse applications

We developed, optimized and validated the process of targeted forward genetics using a gene targeting vector library that contained saturating, random, base pair-substitution mutations within the *ade6* ORF, thus introducing mutations specifically in the Ade6 protein. However, the process is extensible to diverse genomic elements and encoded factors. It can also be implemented using libraries that contain specific (*i.e.*, pre-defined) mutations or any other changes of interest (such as sequential small deletions). A few examples are provided here.

Population-scale, targeted forward genetics supports mutational analyses of structural and regulatory DNA sequence elements, too. It is of particular utility for defining DNA elements whose biological activities require an endogenous, surrounding chromosomal context, such as meiotic recombination hotspots (Wahls and Davidson 2012), long-range regulators of transcription (Catarino *et al.* 2017), and regulators of epigenetic inheritance (Wang and Moazed 2017). One can sprinkle mutations into the candidate regulatory region of interest to identify potential regulatory motifs, and subsequently screen populations of cells with all possible base pair substitutions in the candidate regulatory motifs to define their functional architecture at high resolution (loss of function approach). Reciprocally, in a gain of function approach, one can start with a targeting vector library that contains a stretch of randomized base pairs to discover novel regulatory motifs. The remarkable power of such approaches for identifying and defining novel, biologically important DNA sequence elements at single base-pair resolution has already been validated using more laborious, two-step allele replacement methods (Steiner *et al.* 2011; Steiner *et al.* 2009).

The rationale and approach described for regulatory DNA sequence elements, genes and encoded proteins also applies for analyses of other biomolecules encoded by DNA, such as long non-coding and short regulatory RNAs. In each case, there is considerable latitude in methods used to generate the gene targeting library. Such libraries can be constructed, for example, to have clones that encode individually each possible amino acid substitution in a given protein, to perturb individually and combinatorially G-quadruplex structures, to systematically alter the secondary structure of RNAs, or to modify the sequences of RNAs without affecting their secondary structures.

### Utility of the approach for other model organisms

While this study employed the fission yeast *S. pombe*, targeted forward genetics over long distances could be applied to any organism in which it is possible to carry out gene targeting and that has markers for positive and negative selection. The process is easiest to implement in species that can be propagated as haploids but it is extensible to diploid model organisms, either by carrying out the allele replacements in a heterozygous deletion or by genetic backcrossing.

### Conclusions

The process and workflow described in this study provide a practical approach for precisely targeted, saturating forward genetic screens of discrete genomic elements *in situ*, unencumbered by any additional changes of the target locus or elsewhere in the genome. It is of broad utility for defining the structure and function of DNA sequence elements and their encoded factors within their normal biological context.
